# Analyzing Inter-Leukocyte Communication and Migration *In Vitro*: Neutrophils Play an Essential Role in Monocyte Activation During Swarming

**DOI:** 10.3389/fimmu.2021.671546

**Published:** 2021-05-12

**Authors:** Nicole Walters, Jingjing Zhang, Xilal Y. Rima, Luong T. H. Nguyen, Ronald N. Germain, Tim Lämmermann, Eduardo Reátegui

**Affiliations:** ^1^William G. Lowrie Department of Chemical and Biomolecular Engineering, The Ohio State University, Columbus, OH, United States; ^2^Laboratory of Immune System Biology, National Institute of Allergy and Infectious Diseases, National Institutes of Health, Bethesda, MD, United States; ^3^Max Planck Institute of Immunobiology and Epigenetics, Freiburg, Germany; ^4^Comprehensive Cancer Center, The Ohio State University, Columbus, OH, United States

**Keywords:** neutrophils (PMNs), monocytes, neutrophil swarming, intercellular communication, extracellular vesicles

## Abstract

Neutrophils are known to be the first responders to infection or injury. However, as inflammation progresses, other leukocytes become increasingly important in inflammation propagation, tissue reconstruction, and inflammation resolution. In recent years, there has been an increase in publications that analyze neutrophil behavior *in vitro*, but there remains a gap in the literature for *in vitro* technologies that enable quantitatively measuring interactions between different types of human leukocytes. Here, we used an *in vitro* platform that mimics inflammation by inducing neutrophil swarming to analyze the behavior of various leukocytes in a swarming setting. Using human peripheral blood leukocytes isolated directly from whole blood, we found that myeloid cells and lymphoid cells had different migratory behaviors. Myeloid cells, which are predominately neutrophils, exhibited swarming behavior. This behavior was not seen with lymphoid cells. We perturbed the peripheral blood leukocyte system by adding exogenous leukotriene B_4_ (LTB_4_) to the medium. Notably, only the myeloid cell compartment was significantly changed by the addition of LTB_4_. Additionally, LTB_4_ had no significant impact on myeloid cell migration during the recruitment phase of swarming. To further investigate the myeloid cell compartment, we isolated neutrophils and monocytes to analyze their interaction on the platform. We found that neutrophils increase monocyte migration toward the bioparticle clusters, as measured through speed, chemotactic index, track straightness, and swarm size. These results were confirmed with *in vivo* mouse experiments, where monocyte accumulation only occurred when neutrophils were present. Additionally, we found that both neutrophils and monocytes release the monocyte chemoattractant proteins CCL2 and CCL3 in the presence of *Staphylococcus aureus* bioparticles. Furthermore, extracellular vesicles from swarming neutrophils caused monocyte activation. These findings suggest that neutrophils play an essential role in the onset of inflammation not only by sealing off the site of infection or injury, but also by recruiting additional leukocytes to the site.

## Introduction

Neutrophils are known to be the first responders to infection or injury in the body (1). When neutrophils encounter an inflammatory signal, they undergo a complex, multistep process called neutrophil swarming (2, 3). Neutrophil swarming can be marked by four general phases: scouting, recruitment, equilibrium, and resolution (1–10). During the *scouting* (or lag) phase, neutrophils encounter the chemotactic signal [e.g., pathogen-associated molecular pattern (PAMP) or damage-associated molecular pattern (DAMP)] at the inflammation site through random migration (4). Upon processing the chemotactic signal, neutrophils become activated. This begins the *recruitment* (also called growth or exponential) phase of the swarm, where the activated neutrophils release a myriad of lipid mediators, cytokines, and extracellular vesicles (EVs) that activate and direct the migration of additional neutrophils, thus eliciting exponential growth of the swarm (3, 7). The recruitment phase of the swarm has been characterized by increased neutrophil speed and chemotactic index near the inflammation site (1, 3, 7). When the inflammation site is sealed off from the surrounding tissue, recruitment halts, and the swarm enters the *equilibrium* phase. Neutrophils migrate in and out of the swarm during this stage, but the overall swarm size remains approximately constant. This phase marks pathogen clearance, extracellular matrix digestion, and tissue reconstruction (2, 4). Depending on the size and nature of the inflammation site, the swarm can be resolved within a few hours or persist for days. Over time, other immune cells, especially monocytes and macrophages, become involved in the swarm (1, 4, 10). Monocytes and macrophages have a number of different reported functions, including initiating neutrophil clustering, joining the swarm along the exterior, and clearing away cell debris (including apoptotic neutrophils) (4, 10). Additionally, monocytes and macrophages may have important functions in transitioning the swarm toward resolution (4). Once the pathogens have been cleared from the area (or, in the instance of sterile inflammation, tissue injury repaired), the *resolution* phase begins (11). During the resolution phase, cell debris is cleared away, inflammatory chemokines are degraded, and immune cells either undergo reverse migration or apoptosis (4), though this phase is often not explored in detail in swarming studies (1, 3, 5–8).

Neutrophil swarming has been studied *in vivo* and *in vitro* (4). *In vivo* studies are usually performed in zebrafish and mouse models and have irreplaceable value for studying inflammation in the complex environment of tissue (2, 10, 12, 13). Previous *in vivo* studies have shown neutrophil migration in various tissues and provided the first experimental proofs of neutrophil swarming (1, 2, 14, 15). Studies in zebrafish have elucidated neutrophil signaling pathways (16, 17), phagocytic capabilities (13), resolution mechanisms (13, 18), immunodeficiency models (12, 19), and pathogen interactions in specific disease settings (12, 20). Though *in vivo* studies have been critical to reach our current understanding of the immune system, *in vitro* studies allow the direct study of human neutrophils and provide experimental advantages that make *in vitro* studies essential complements to *in vivo* studies. *In vitro* studies, though simplified, excel in their high-throughput nature, high reproducibility, tight control of experimental variables, and direct access to cell supernatant for analyzing secreted mediators (3, 6, 21). Previous *in vitro* studies have investigated human neutrophil migration in response to specific chemotactic gradients (22–24) and analyzed the molecular content released by swarming human neutrophils in detail (3, 7). In recent years, there has been an increase in publications describing neutrophil behavior in detail in both *in vivo* and *in vitro* systems (2, 3, 6, 7, 10, 16, 25–27), but there remains a gap in the literature that explores in depth the relationship and intercellular communication between neutrophils and other leukocytes involved in the inflammation response.

Here, we expanded our *in vitro* technology that we have previously used to analyze neutrophil swarming (3, 6, 7, 28) to incorporate multiple types of leukocytes, enabling the analysis of intercellular communication between leukocyte types. We investigated the interaction of myeloid and lymphoid cells present in the natural mixture of peripheral blood leukocytes (PBLs) found in human blood. While myeloid cells induced some lymphoid cell activation, only myeloid cells exhibited swarming behavior. Furthermore, we found that neutrophils elicited monocyte swarming, though monocytes did not swarm on their own. Investigation into the mechanism of monocyte activation indicated that swarming neutrophil EVs (snEVs) participate in monocyte activation. Our results suggest that neutrophils are essential to the start of the complex immunocascade.

## Materials and Methods

### Microarray Device Fabrication

Standard lithography procedures were used to create a polydimethylsiloxane (PDMS) microstamp as previously reported (6). Briefly, a 40-µm layer of SU-8 2050 (Kayaku Advanced Materials, Westborough, MA) was spun onto a silicon wafer (University Wafers, South Boston, MA). Then, the wafer was exposed to UV light through a chrome mask to crosslink SU-8 in the desired pattern, and unexposed SU-8 was subsequently removed with photoresist developer. A 10:1 ratio of PDMS and its curing agent (Dow, Midland, MI) was poured over the wafer, vacuum treated to remove air bubbles, and cured for 6 hours at 65°C. The cured PDMS was then cut around the pattern and carefully peeled off the wafer to yield arrays of posts with the following dimensions: 30-µm diameter, 500-µm center-to-center spacing, and 40-µm tall.

The microarray device was fabricated through a microstamping procedure (7). **Figure Supplementary 1** shows the details. Briefly, 1.6 mg/mL Zetag 8185 (BASF, Ludwigshafen, Germany) was spun onto a clean glass slide. The aforementioned PDMS array was incubated on the Zetag layer for 20 min with a balanced weight (3.8 g/cm^2^) placed on the back of the stamp. The stamp was then placed on a clean glass slide and incubated for 10 min under the balanced weight while the Zetag solution transferred to the glass slide. The glass slide with the Zetag pattern was then dried overnight at room temperature. Then, a 16-well chamber (Grace Bio-Labs, Bend, OR) was secured to the top of the slide. Then, 100 µL of 67 µg/mL solution of *Staphylococcus aureus* (Wood strain without protein A) bioparticles conjugated to AlexaFluor 594 (ThermoFisher, Waltham, MA) was incubated in the wells for 20 min on a rocker at room temperature. The bioparticles adhere to the Zetag spots through electrostatic interaction. After incubation, excess bioparticles were removed by washing thoroughly under a strong stream of DI water. This yields an array of bioparticle clusters, which are targets for neutrophil swarming. The device can then be stored for up to 3 months at 4°C in a dust-free environment.

On the day of the experiment, the glass surface of the device was coated with 10 ng/mL human fibronectin (ThermoFisher) in fetal bovine serum (FBS) for 1 h at room temperature. The surface was washed 3x with phosphate buffered saline (PBS) for 5 min each. The swarming assay can be run without the fibronectin coating, but the protein layer improves cell adhesion to the glass, which is necessary for cell fixation experiments. The device surface was kept wet with PBS until the cell suspension was prepared. 200 μL of the prepared cell suspension was added to a well of the device.

### Control Device Fabrication

Many of our experiments required conditions where no bioparticle microarray is present (i.e., non-activated cells). For these experiments, the 16-well imaging spacer was secured to a clean glass slide. On the day of the experiment, the glass surface of the device was coated with 10 ng/mL human fibronectin in FBS for 1 h at room temperature. The surface was washed 3x with PBS for 5 min each. The device surface was kept wet with PBS until the cell suspension was prepared. 200 μL of the prepared cell suspension was added to a well of the device. This device was used for the control conditions of the immunostaining experiment and all conditions of the snEV experiment.

### Leukocyte Preparation

Human blood was collected in K2-EDTA tubes according to protocol #2018H0268 approved by the Biomedical Sciences Committee Institutional Review Board (IRB) at The Ohio State University. For experiments using the PBLs, the red blood cells (RBCs) were depleted through RBC lysis (29). A cell lysis buffer of 150 mM ammonium chloride, 10 mM sodium bicarbonate, and 0.1 mM EDTA at 7.4 pH was prepared. Blood was added to the lysis buffer at a 1:20 ratio and incubated for 5 min at RT. Then, the mixture was centrifuged at 350 x g for 5 min and the supernatant aspirated. The pellet was resuspended into lysis buffer (1/5 of the previous volume), incubated for 5 min, and centrifuged at 350 x g for 5 min to remove residual RBCs and debris. At this stage, the leukocytes were >99% pure, calculated as the ratio of the nucleated cell count to the total cell count (data not shown). The leukocytes were resuspended in Iscove’s Modified Dulbecco’s Medium (IMDM, ThermoFisher) and stained with 0.5 μM CellTracker Green (CTG, ThermoFisher) for 45 min and 20 μg/mL Hoechst 33342 (ThermoFisher) for 10 min. Then PBS was added to 5 times the original volume, the solution was centrifuged at 350 x g for 5 min, and the supernatant was aspirated. Finally, the leukocytes were resuspended in IMDM with 20% FBS to a concentration of 0.6 million cells/mL.

### Neutrophil Preparation

Human blood was collected, and the leukocytes were enriched according to the above RBC lysis procedure. Then, neutrophils were isolated from the leukocyte mixture using an EasySep™ Human Neutrophil Isolation Kit (STEMCELL Technologies, Vancouver, Canada), which enriches neutrophils through negative-selection immunomagnetic labeling and subsequent magnetic separation. Neutrophils were resuspended in IMDM with 20% FBS and stained with CTG and Hoechst 33342 as described above. Neutrophils were adjusted to a concentration of 0.6 million cells/mL for swarming experiments of neutrophils alone and neutrophils with monocytes.

### Monocyte Preparation

Human blood was collected as described above. Monocytes were isolated using an EasySep™ Direct Human Monocyte Isolation Kit (STEMCELL Technologies), which uses a series of negative-selection immunomagnetic labeling and subsequent magnetic separations to isolate monocytes directly from human whole blood. Then, monocytes were resuspended in IMDM with 20% FBS and stained with 0.25 μM CellTracker deep red (CTDR, ThermoFisher) for 45 min and 20 μg/mL Hoechst 33342 for 10 min. Monocytes were adjusted to a concentration of 0.6 million cells/mL for swarming experiments of monocytes alone and monocytes with neutrophils.

### LTB_4_ Addition to Prepared PBL Suspensions

Leukotriene B_4_ (LTB_4_) was added to the PBL suspensions at 100 nM. Initially, the LTB_4_ was present as a 0.1 mg/mL solution in ethanol (Cayman Chemical, Ann Arbor, MI). The proper amount of LTB_4_ solution was added to an empty microtube through a serial dilution using pure ethanol (ThermoFisher). The solution was desiccated under vacuum for >20 min to ensure complete evaporation of the ethanol. Then, 20 μL of PBS was added to the desiccated LTB_4_ and given 10 min to equilibrate. Next, 500 μL of cell suspension was added and given 5 min to equilibrate. The cell suspension was then added onto the prepared devices and the time-lapse experiments were started immediately.

### *In Vivo* Studies

*DsRed^+/-^ Cx3cr1^gfp/gfp^ Tyr^c-2J/c-2J^* mice were crossed from individual mouse strains that were purchased from Jackson Laboratories (JAX006051, JAX005582, JAX000058). In previous work, the Germain lab has compared *CAG-DsRed Cx3cr1^gfp/+^ Tyr^c-2J/c-2J^* and *CAG-DsRed+/- Cx3cr1^gfp/gfp^ Tyr^c-2J/c-2J^* mice and found similar monocyte dynamics for both genotypes (1). We selected *Cx3cr1^gfp/gfp^* mice for these experiments to achieve optimal detection of the weak GFP fluorescence in this monocyte/macrophage subset, as *Cx3^cr1gfp/+^* mice often yielded poorer quality images. Mice were maintained in specific-pathogen-free conditions at an Association for Assessment and Accreditation of Laboratory Animal Care-accredited animal facility at the NIAID, National Institutes of Health, and were used under a study protocol from Dr. Ron Germain approved by NIAID Animal Care and Use Committee (National Institutes of Health).

Two-photon intravital imaging of ear pinnae of anaesthetized mice and laser-induced tissue injury was performed as previously described (1). Mice were anaesthetized using isoflurane (Baxter; 2% for induction, 1–1.5% for maintenance, vaporized in an 80:20 mixture of oxygen and air) and placed in a lateral recumbent position on a custom imaging platform such that the ventral side of the ear pinna rested on a coverslip. A strip of Durapore tape was placed lightly over the ear pinna and affixed to the imaging platform to immobilize the tissue. Images were captured towards the anterior half of the ear pinna where hair follicles are sparse. Images were acquired using an inverted LSM 510 NLO multiphoton microscope (Carl Zeiss Microimaging) enclosed in a custom-built environmental chamber that was maintained at 32°C using heated air. This system had been custom fitted with three external non-descanned photomultiplier tube detectors in the reflected light path. Images were acquired using a 25X/0.8 numerical aperture (NA) Plan-Apochromat objective (Carl Zeiss Imaging) with glycerol as the immersion medium. Fluorescence excitation was provided by a Chameleon XR Ti : Sapphire laser (Coherent) tuned to 920nm for excitation of both DsRed and eGFP. For four-dimensional data sets, three-dimensional stacks were captured every 30 s. Mice were on the *Tyr^c-2J/c-2J^* (B6.Albino) background to avoid laser-induced cell death of light-sensitive skin melanophages.

To study neutrophil swarming within interstitial tissue, mice were anaesthetized with isoflurane and underwent a brief skin trauma to recruit neutrophils from the circulation to the dermal interstitium. The anesthetized mouse was placed on a scale and 30N per cm^2^ pressure was applied for 15–20 s on the mouse ear with the investigator’s thumb. For neutrophil depletion, mice were injected intraperitoneally with 100 µg/ml anti-Gr1 (clone RB6-8C5, BioXCell) 24 hours before applying brief skin trauma. We opted to use anti-Gr1 rather than anti-Ly6G (1A8) because we found that some neutrophils remained after depletion with anti-Ly6G (1A8) (data not shown). As a few neutrophils can set off the cascade of myeloid cell swarms, it was crucial to choose conditions of complete neutrophil absence, which we achieved with anti-Gr1 mediated depletion. Three hours after brief skin trauma, mice were prepared for skin imaging as described above and rested in the heated environmental chamber for 30–60 min before the first focal tissue damage was induced. For focal tissue damage in the ear dermis, the Chameleon XR Ti:sapphire laser (Coherent) was tuned to 850nm and the laser intensity adjusted to 80 mW. At pixel dimensions of 0.14 X 0.14 µm, a circular region of interest of 25–35 µm in diameter (approximately 1–2 X 10^-6^ mm^3^ in volume) was defined in one focal plane, followed by laser scanning at a pixel dwell time of 0.8 µs for 35–50 iterations, depending on the tissue depth of the imaging field of view. Immediately after laser-induced tissue damage, imaging of the neutrophil response was started at typical voxel dimensions of 0.72 X 0.72 X 2 µm.

### *In Vitro* Immunofluorescence

Neutrophil and monocyte suspensions were prepared and added to the prepared device (either with or without a bioparticle array, according to the condition) as described above. The cells were incubated on the device for 60 min before fixation. Cells were fixed in 4% formaldehyde for 15 minutes at RT. Then, cells were permeabilized in ice-cold methanol for 10 minutes at -20°C. Cells were incubated with TSA blocking buffer (ThermoFisher) with 0.3% Triton-X 100 overnight at 4°C. Then, a mixture of 5 µg/mL rabbit-anti human CCL2 antibody (Abcam) and 8 µg/mL mouse-anti human CCL3 (R&D Systems) in blocking buffer + 0.3% Triton-X 100 was added to the slide and incubated for 2 h at RT. Then the sample was blocked with 5% goat serum + 0.3% Triton-X 100 for 1 h at RT. Finally, a mixture of goat anti-rabbit IgG AlexaFluor 488-conjugated (ThermoFisher, 500x dilution) and goat anti-mouse IgG AlexaFluor 647-conjugated (ThermoFisher, 500x dilution) in blocking buffer + 0.3% Triton-X 100 was incubated on the sample for 1 h at RT. The sample was mounted with ProLong Gold Antifade Mountant with DAPI (ThermoFisher), sealed with nail polish, and stored at 4°C until imaging. The following controls were considered on devices with and without a bioparticle array: IgG controls (mouse and rabbit) and secondary-only staining.

### snEV Preparation and Addition to Monocytes

Swarming neutrophil extracellular vesicles (snEVs) were generated and enriched as previously described (7). Briefly, neutrophils were incubated in IMDM + 2.5% EV-depleted FBS on an array of *S. aureus* bioparticles. A geometry of 30-µm diameter, 200-µm center-to-center spacing was used to obtain a sufficient density of EVs from actively swarming neutrophils. After 45 min, the supernatant was collected and filtered with a 2-µm filter to remove any cell debris above that size. Free mediators were removed from the system through tangential flow filtration and diafiltration, yielding purified snEVs. The purified snEVs were concentrated to 10^10^ snEVs/mL using a 3 kDa centrifugal filter (Amicon). After processing, snEVs were characterized using tunable resistive pulse sensing (TRPS). The concentration and size distribution of small EVs (70 to 420 nm) was measured using an NP150 membrane on the qNano Gold (Izon Science).

Monocytes were isolated as described above and suspended in IMDM + 20% FBS at 0.6 mil cells/mL. Then, 1000 snEVs/cell were added to monocytes (12 µL concentrated snEVs: 1 mL monocytes). For the control condition, 12 µL PBS was added instead of snEVs. 200 µL monocytes were added to a control device (preparation described above) and the imaging experiment was started immediately.

### Cell Imaging and Analysis

200 μL of the prepared cell suspension was added to a well of the prepared device. The device was then loaded onto a fully automated Ti2 microscope (Nikon, Tokyo, Japan) equipped with a stage incubator (Okolab, Pozzuoli, Italy) set at 37°C and 5% CO_2_. Time-lapse images recorded leukocyte migration on the bioparticle devices on appropriate fluorescent and bright field channels at a maximum frame rate of 1 frame every 10 seconds to achieve accurate cell tracking. Fluorescence images were captured using the following filters: 405 nm (DAPI, Hoechst), 488 nm (CTG, AlexaFluor 488), 594 nm (AlexaFluor 594), and 647 nm (CTDR, AlexaFluor 647).

Cell tracks were generated using Imaris spot detection and tracking (Bitplane, Zürich, Switzerland). Spots were detected using the cytoplasmic dye (i.e., CTG or CTDR) using a spot radius of 8 μm, the approximate radius of a leukocyte. Tracking was performed in autoregression motion mode with a maximum gap size of 3 frames, maximum distance travelled of 15 μm/frame, and minimum track length of 120 s. In experiments using PBLs, myeloid cells were distinguished from lymphocytes according to the ratio of the intensity of CTG and Hoescht 33342 fluorescence channels, as described in the results section. Chemotactic index (CI) was calculated from the position and velocity data in the cell tracks. The velocity vector is obtained through the velocity angle generated by Imaris. The position vector is calculated manually from the X, Y position of the cell and the X, Y position of the nearest bioparticle target. The cosine of the angle between these vectors is the CI (**Figure Supplementary 2**). Radial velocity was calculated as the CI multiplied by the speed of the cell.

The Analyze Particles function available through ImageJ (30) was used to analyze 2D swarm size, immunofluorescence, and monocyte shape descriptors. Briefly, background fluorescence intensities were subtracted from the images. Then, the images were duplicated, and an intensity threshold was applied to create binary images. For 2D swarm size, the size of the swarms was calculated as the 2D area of the binary particles that covered the targets. The size was tracked over each frame to generate a growth curve of the swarm over time. For immunofluorescence data, the binary images were redirected to the background-subtracted images and analyzed by cell. The average immunofluorescence from the isotype controls was subtracted from the results. For the monocyte activation by snEVs experiment, the shape descriptors of circularity and area were obtained through the Analyze Particles function after image thresholding.

### Data and Statistical Analysis

Here, data are presented as mean ± SD unless otherwise noted. The statistical tests used in this paper were student t-tests with α = 0.05 unless otherwise noted.

## Results

### Distinguishing Myeloid and Lymphoid Cells Among PBLs

**Figure 1:**
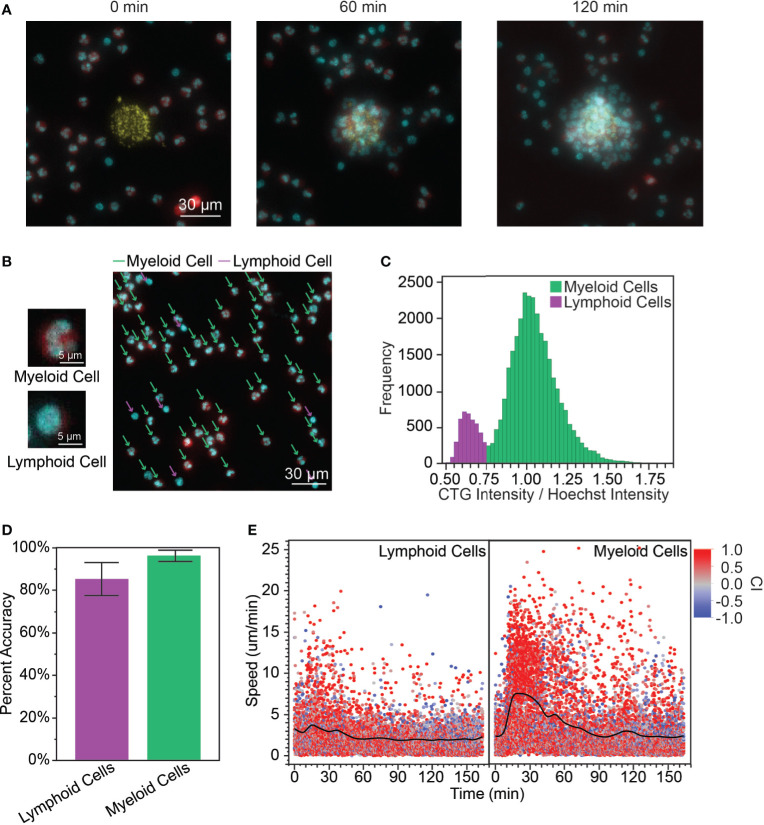
**(A)** When the PBLs were first added to the array of *S. aureus* bioparticle targets, the cells were dispersed randomly across the surface of the device (0 min). A cluster (or swarm) of PBLs grows around the bioparticle target over time (60 min, 120 min). (yellow: bioparticle target, red: PBL cytoplasm, cyan: PBL nucleus, scale bar: 30 µm) **(B)** Myeloid cells and lymphoid cells had distinct nuclear morphology. Myeloid cells had polymorphonuclei while lymphoid cells had large, circular nuclei and relatively little cytoplasm. The cytoplasm stain used for this experiment was CTG and the nuclear stain was Hoechst. The CTG channel was artificially colored red and Hoechst cyan to facilitate visualization. [scale bars: 5 µm (left), 30 µm (right)] **(C)** Calculating the ratio of intensities (RI) of the cytoplasm to nuclear stains yielded a bimodal distribution, which was used to distinguish myeloid and lymphoid cells. **(D)** The bimodal distribution of the RI correctly identified myeloid and lymphoid cells with high accuracy (96.4 ± 2.7% for myeloid cells and 85.4 ± 7.8% for lymphoid cells, average of n = 172 leukocytes/donor, N = 3 donors). **(E)** The myeloid cells followed a similar speed pattern to isolated neutrophils, with increased speed during the recruitment phase of the swarm (7.32 ± 4.70 µm/min for t = 15 – 30 min, n = 1149; compared to 2.91 ± 2.75 µm/min for t = 0 – 12 min, n = 1114, p < 0.0001). Lymphoid cells exhibited only a slight increase in speed during this phase. (3.31 ± 2.86 µm/min for t = 15 – 30 min, n = 906; compared to 2.94 ± 2.42 µm/min t = 0 – 12 min, n = 909, p = 0.0027). There was no significant difference between myeloid and lymphoid cell speed during the scouting phase, while myeloid cells had significantly higher speed than lymphoid cells during the recruitment phase (p = 0.42 and p < 0.0001 for t = 0 – 12 min and t = 15 – 30 min, respectively).

Upon adding the peripheral blood leukocytes (PBLs) to our *in vitro* microarray platform, we observed swarming-like behavior among the PBLs (**Figure 1A**, **Video S1**), similar to neutrophil swarming as previously reported (1, 3, 7). First, we considered whether we could use differences in myeloid and lymphoid cell morphology to distinguish the migration patterns of myeloid and lymphoid cells in the PBLs without complex isolation and staining procedures. Myeloid cells have either multilobed nuclei (e.g., neutrophils, eosinophils, and basophils, **Figure 1B**, top) or kidney-shaped nuclei (e.g., monocytes), while lymphoid cells (e.g., T cells, B cells, and NK cells) have large, circular nuclei (**Figure 1B**, bottom) (31). Although these cells are simple to distinguish by eye (**Figure 1B** right), it is challenging to distinguish these cells by nuclear shape through image recognition software. Therefore, we used the principle that lymphoid cells tend to have large nuclei and very little cytoplasm, while myeloid cells have comparatively higher cytoplasm to develop a method for distinguishing them. We defined the ratio of the intensity (RI) of the cytoplasm stain to the intensity of the nuclear stain, normalized by the average ratio at the given time point, as a feature that has a bimodal distribution (**Figure 1C**). We defined a cutoff (CO) at the minimum frequency in this distribution and assigned cells with RI ≥ CO as myeloid cells and RI < CO as lymphoid cells. In the presented data, CO = 0.75, but this varied from donor to donor. We validated this prediction by manually classifying a random sample of PBLs based on nuclear morphology and comparing that to the prediction using the RI. The percent accuracy was calculated object-wise, defined as the true positive rate of classified cells (i.e., number of myeloid cells correctly classified/total number of myeloid cells, etc.) (**Figure 1D**). Using this cutoff, we calculated the myeloid to lymphoid cell ratio (MLR) for our various donors. The mean MLR was 1.61 ± 1.07 and the range was 0.863 – 3.47 (**Supplementary Table 1**).

Since most myeloid cells are neutrophils, we expected the migratory behaviors of myeloid cells on our platform to be generally representative of neutrophil migratory behaviors as described in the introduction. This was corroborated by looking at the speed of myeloid cells over time (**Figure 1E**). The first myeloid cells encounter the bioparticle target through random migration during a brief scouting phase. This is followed by a sharp rise in cell speed that is characteristic of the recruitment phase where neutrophil-generated chemoattractants create a chemoattractant gradient to direct neutrophil migration toward the inflammation site, as previously shown in *in vitro* neutrophil studies (3, 7). Interestingly, lymphocytes also exhibited increased speed during the recruitment phase of the swarm, though this increase was less pronounced. There was no significant difference between the speed of myeloid and lymphoid cells during the scouting phase, but myeloid cells had significantly higher speed compared to lymphoid cells during the recruitment phase.

### Adding an Artificial Source of LTB_4_ Affects the Dynamics of Leukocyte Swarming

**Figure 2:**
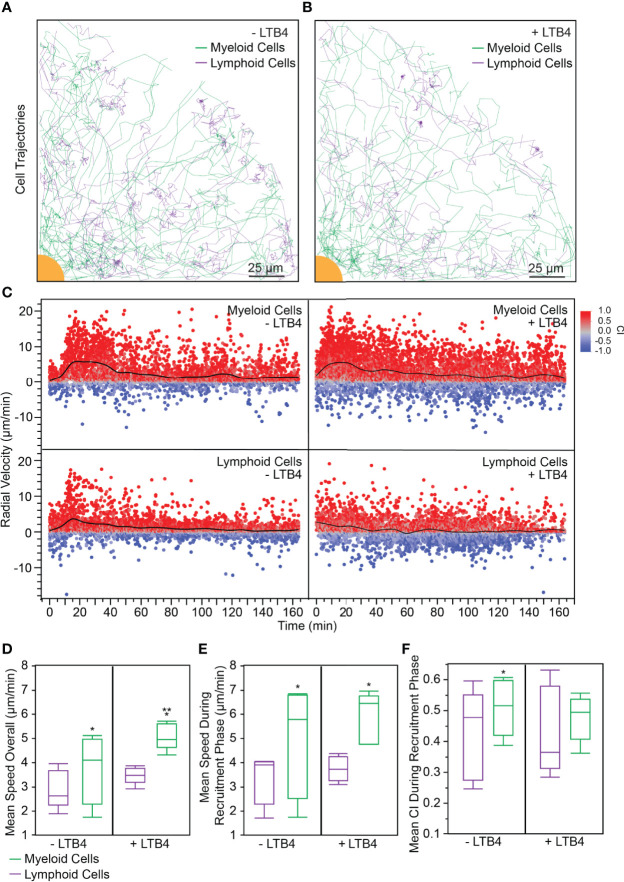
**(A)** 20% of myeloid and lymphoid tracks (randomly selected) from a representative experiment are shown. In the absence of exogenous LTB_4_, myeloid cells followed more radial tracks toward the target. Interestingly, few lymphoid cell tracks came within the swarm. (scale bar: 25 µm) **(B)** 20% of myeloid and lymphoid tracks (randomly selected) from a representative experiment are shown. When exogenous LTB_4_ was added to the system, myeloid cells followed less direct paths to the target, though a swarm still formed. Few lymphoid cells entered the swarm. (scale bar: 25 µm) **(C)** Myeloid cells without exogenous LTB_4_ followed the expected swarming pattern with scouting (t = 0 – 15 min), recruitment (t = 15 – 45 min), and equilibrium (t > 45 min) phases. The recruitment phase had a significantly higher radial velocity than either the scouting or equilibrium phase (Anova, p < 0.0001). When exogenous LTB_4_ was present, the scouting phase was not distinguishable, because the myeloid cells were already activated by the LTB_4_ (Anova between t = 0 – 15 min and t = 15 – 45 min, α = 0.05). Interestingly, lymphoid cells had a small phase of recruitment toward the swarm, indicated by the increased radial velocity from 15 – 45 min (Anova between t = 0 – 15 min and t = 15 – 45 min, p < 0.05). When exogenous LTB_4_ was present, this recruitment phase was not observed (Anova between t = 0 – 15 min and t = 15 – 45 min, α = 0.05). **(D–F)**. Statistical tests: *p < 0.05 for student t-test comparing myeloid to lymphoid cells within a given LTB_4_ condition (either $LTB_{4}^{-}$ or $LTB_{4}^{+}$). **p < 0.05 for student t-test comparing the $LTB_{4}^{-}$ to the $LTB_{4}^{+}$ condition within a given cell type (either myeloid or lymphoid cells). **(D)** The mean speed of myeloid cells throughout the experiment was higher than that of lymphoid cells, regardless of the presence of exogenous LTB_4_ ($LTB_{4}^{-}$ condition: 3.72 ± 1.41 µm/min and 2.90 ± 0.79 µm/min, for myeloid and lymphoid cells respectively, p = 0.04, N = 5. $LTB_{4}^{+}$ condition: 5.08 ± 0.55 µm/min and 3.47 ± 0.35 µm/min, for myeloid and lymphoid cells respectively, p = 0.007, N = 5). Exogenous LTB_4_ caused increased speed of myeloid cells but did not significantly impact lymphoid cell speed (α = 0.05). **(E)** Similarly, the mean speed of myeloid cells during the recruitment phase of the experiment was higher than that of lymphoid cells, regardless of the presence of exogenous LTB_4_ ($LTB_{4}^{-}$ condition: 4.89 ± 2.26 µm/min and 3.31 ± 1.02 µm/min, for myeloid and lymphoid cells respectively, p = 0.02, N = 5. $LTB_{4}^{+}$ condition: 5.90 ± 1.05 µm/min and 3.75 ± 0.51 µm/min, for myeloid and lymphoid cells respectively, p = 0.005, N = 5). However, there was no significant difference between myeloid or lymphoid cell speed during the recruitment phase of the experiment between $LTB_{4}^{+}$ and $LTB_{4}^{-}$ conditions (α = 0.05). **(F)** Without exogenous LTB_4_, myeloid cells had a significantly higher CI during the recruitment phase of the swarm compared to lymphoid cells (0.51 ± 0.09 and 0.43 ± 0.14, respectively, p = 0.04, N = 5). There was no significant difference between myeloid and lymphoid cells with exogenous LTB_4_ added (0.48 ± 0.07 and 0.43 ± 0.14, respectively, α = 0.05, N = 5).

We perturbed the PBLs with 100 nM LTB_4_ to analyze the effect of exogenous LTB_4_ on swarming and to test our platform’s ability to capture differences between a perturbed and control system. Interestingly, leukocyte swarms formed around the bioparticle targets regardless of the presence of exogenous LTB_4_ (**Video S2**), which speaks to the highly controlled, robust nature of the swarming response. Mapping the cell trajectories of myeloid and lymphoid cells demonstrated that myeloid cells converged on the bioparticle target both in the presence and in the absence of LTB_4_. Interestingly, most of the cells that reached the target were myeloid cells, while the lymphoid cells tended to accumulate around the edge of the swarm (**Figures 2A**, **B**).

We also analyzed the radial velocity of myeloid and lymphoid cells over time. The condition where no exogenous LTB_4_ was added $\left(LTB_{4}^{-}\right)$ showed the standard phases of swarming in the myeloid cell compartment: a scouting phase (with a low mean radial velocity), a recruitment phase (where the mean radial velocity increased around 15 min), and an equilibrium phase (where the mean radial velocity tapers off after 45 min) (**Figure 2C** top left), similar to previously reported neutrophil behavior (3). However, when exogenous LTB_4_ was added $\left(LTB_{4}^{+}\right)$ the scouting phase was negligible, with the radial velocity nearing its maximum within five minutes (**Figure 2C** top right). Lymphoid cells exhibited a brief recruitment phase where the mean radial velocity increased in the $LTB_{4}^{-}$ condition (**Figure 2C** bottom left), though the cell trajectories suggested that some of these recruited cells did not enter the swarm. In the $LTB_{4}^{+}$ condition, in contrast, the radial velocities of lymphoid cells remained relatively constant throughout the experiment (**Figure 2C** bottom left). Furthermore, we analyzed the bulk average of various parameters for myeloid cells and lymphoid cells in $LTB_{4}^{-}$ and $LTB_{4}^{+}$ conditions across 5 donors. The first parameter we analyzed was mean speed overall, which was calculated as the average cell speed across the entire experiment (t=0 to t=180 min). In both $LTB_{4}^{-}$ and $LTB_{4}^{+}$ conditions, the myeloid cells had significantly higher speed than the lymphoid cells (**Figure 2D**). Furthermore, the myeloid cells had a significantly higher mean speed overall in the $LTB_{4}^{+}$ condition than in the $LTB_{4}^{-}$ condition (**Figure 2D**), which suggests that the exogenous LTB_4_ caused additional activation of myeloid cells outside of the expected swarming response. Second, we analyzed the average cell speed during the recruitment phase of swarming, where the recruitment phase was defined as the period of increased myeloid cell speed (**Figure 2E**). While myeloid cells still exhibited a higher speed than lymphoid cells for each condition, there was no significant difference between the average myeloid cell speed between $LTB_{4}^{-}$ and $LTB_{4}^{+}$ conditions during the recruitment phase. Finally, we analyzed the mean CI during the recruitment phase. In the $LTB_{4}^{-}$ condition, the myeloid cells had a significantly higher CI than the lymphoid cells (**Figure 2F**). Interestingly, the $LTB_{4}^{+}$ condition did not have a significant difference between myeloid and lymphoid cell CI, which suggests that the presence of exogenous LTB_4_ attenuates the precision of the swarming response, though the myeloid cells are still able to form stable swarms. Furthermore, there was no significant change in any of these three parameters for the lymphoid cells between $LTB_{4}^{-}$ and $LTB_{4}^{+}$ conditions, which suggests that LTB_4_ does not directly affect lymphoid cell migration and activation.

### Neutrophils Enhance Monocyte Migration During *In Vitro* Swarming

**Figure 3:**
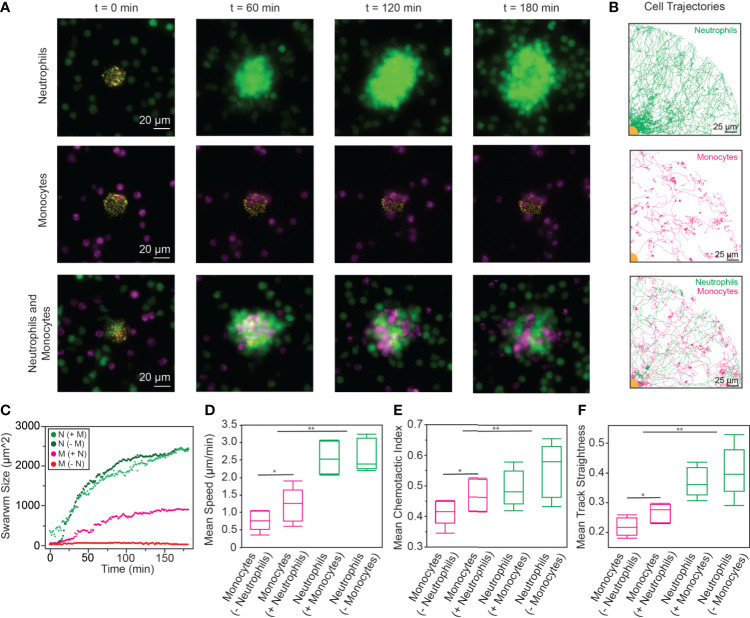
**(A)** Isolated neutrophils swarmed on the *S. aureus* bioparticle targets over time (top). However, when monocytes were isolated and added to the targets, no swarming was observed (middle). Interestingly, when neutrophils and monocytes were mixed and added to the bioparticle targets, both neutrophils and monocytes swarmed toward the target (bottom). (scale bars: 20 µm) **(B)** Neutrophils alone had relatively radial trajectories toward the bioparticle target. Monocytes alone exhibited random migration, with no discernable swarming. When the cells were added in concert, the monocyte trajectories became more radial as the cells were recruited to the swarm. (scale bars: 25 µm) **(C)** Neutrophils followed similar recruitment patterns to the swarm regardless of the presence of monocytes, measured by 2D swarm size (neutrophils (- monocytes): 2320 ± 590 µm^2^, neutrophils (+ monocytes): 2290 ± 730 µm^2^, p = 0.24, n = 527 each). Monocytes, on the other hand, exhibited dramatically increased recruitment to the swarm in the presence of neutrophils (monocytes (- neutrophils): 8 ± 41 µm^2^, monocytes (+ neutrophils): 870 ± 530 µm^2^, n = 527, p < 0.0001). Monocyte accumulation was lower than neutrophil accumulation in each condition (p < 0.0001). **(D–F)**. Monocytes exhibited increased speed, CI, and track straightness in the presence of neutrophils (Statistical test: *p < 0.05, student t-test between monocytes (- neutrophils) and monocytes (+ neutrophils), N = 4). There was no significant difference between the speed, CI, or track straightness of neutrophils in the presence of monocytes and neutrophils alone (α = 0.05, student t-test between neutrophils (- monocytes) and neutrophils (+ monocytes), N = 4). Additionally, neutrophils exhibited higher speed, CI, and track straightness than monocytes [Statistical test: **p < 0.05, student t-test between all monocyte data (+/- neutrophils) and all neutrophil data (+/- monocytes)].

Since the myeloid cells were the primary effector cells on our platform, we wanted to investigate the myeloid cell compartment more thoroughly. We isolated neutrophils and monocytes independently from the same donor and stained them separately. We added neutrophils and monocytes independently to our platform, as well as adding a mixture of neutrophils and monocytes. In all three cases, cells accumulated on the bioparticle targets over time, though only the conditions where neutrophils were present exhibited swarming behavior (**Figure 3A**, **Video S3****-****S5**). We tracked cell recruitment toward the bioparticle targets. Interestingly, while monocyte migration when no neutrophils were present appeared largely random, monocyte tracks when neutrophils were present appeared more radial, similar to how neutrophil tracks appear during swarming (**Figure 3B**). To quantify the recruitment of cells toward the swarms, we tracked the 2D swarm size over time (**Figure 3C**). Similar to what previous publications have shown (3, 6), neutrophils exhibited a recruitment phase before the swarm size leveled out during the equilibrium phase. When monocytes were added to the platform alone, they became activated upon reaching the bioparticle targets and adhered to the bioparticles (**Video S4**) but did not follow the same exponential growth that is indicative of the recruitment phase in a neutrophil swarm. Monocyte behavior varied between donors, and one donor demonstrated swarming-like behavior in the monocytes alone condition (**Figure Supplementary 3**). Interestingly, when neutrophils and monocytes were both added to the platform, the monocytes occupied a significantly higher area around the target and had more of a recruitment phase than monocytes that were alone. In the case where neutrophils and monocytes were present at equal concentrations, there was significantly more neutrophil recruitment than monocyte recruitment. Additionally, we quantified cell migration by looking at mean cell speed, CI, and track straightness for each condition across 4 donors (**Figures 3D–F**). Interestingly, the presence of monocytes did not significantly alter neutrophil migration across any of the parameters we measured. However, the presence of neutrophils impacted the migration of monocytes, causing a significant increase in mean speed, CI, and track straightness when neutrophils were present. There was also a significant difference between neutrophil and monocyte migration across all conditions.

### Neutrophils Enhance Monocyte Migration During *In Vivo* Swarming in a Mouse Model

**Figure 4:**
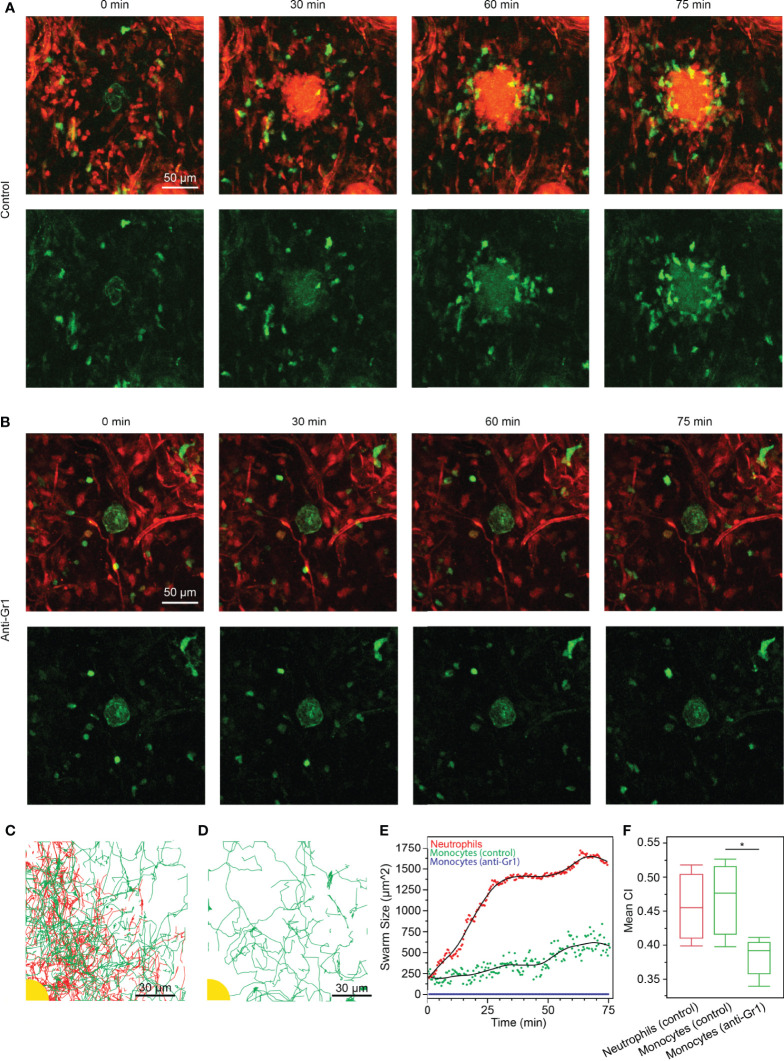
**(A, B)** Time-lapse sequences of endogenous neutrophil and macrophage/monocyte dynamics in *DsRed^+/-^ Cx3cr1^gfp/gfp^ Tyr^c-2J/c-2J^* mice (macrophages/monocytes in green, stroma and neutrophils in red). (scale bars: 50 µm) **(A)** As previously shown (1), small red cells (neutrophils) cluster within 30 min around the local site of laser-induced tissue injury (green autofluorescence in the image center). Cx3cr1-GFP macrophages/monocytes assemble around neutrophil cluster with a time delay. Representative experiment of n=3. **(B)** Anti-Gr1 mediated treatment of mice depleted neutrophils from mouse tissue (macrophages/monocytes in green, stroma in red), resulting in non-directional motility of Cx3cr1-GFP cells around the tissue lesion site. Representative experiment of n=2. **(C)** Tracks of neutrophils (red) and monocytes (green) as they accumulated around the site of injury (yellow). (scale bar: 30 µm) **(D)** Tracks of monocytes (green) when neutrophils were depleted with anti-Gr1. No accumulation was observed around the site of injury (yellow). (scale bar: 30 µm) **(E)** Neutrophil swarming occurred rapidly around the site of injury and leveled out after 30 min at 1400 ± 35 µm^2^. In the presence of swarming neutrophils, monocytes accumulated gradually over time, reaching 542 ± 114 µm^2^ after 75 min. Conversely, no monocyte accumulation occurred when neutrophils were depleted. **(F)** The CI of monocytes was significantly higher in the control experiment than when neutrophils were depleted with anti-Gr1 (Statistical test: *p < 0.005). There was no significant difference between the CI of neutrophils and monocytes in the control experiment (α = 0.05).

To confirm our findings from the *in vitro* swarming platform in a physiological setting, we studied the response of swarming neutrophils and macrophages/monocytes in the ear skin tissue of living anesthetized mice. By performing two-photon intravital microscopy on mildly inflamed dermis of *DsRed^+/-^ Cx3cr1^gfp/gfp^ Tyr^c-2J/c-2J^* (B6.Albino) mice, we could previously visualize neutrophils and Cx3cr1-GFP positive macrophages/monocytes side-by-side in the same tissue during their response to a small focal, laser-induced tissue injury (1). It should be noted that here we refer to a Cx3cr1-GFP positive monocyte/macrophage population, which is distinct from the blood-recruited inflammatory CCR2+ monocytes, which are Cx3cr1-GFP^low/-^. In agreement with our previous results, we observed that neutrophils and Cx3cr1-GFP positive myeloid cells had different dynamic behavior: neutrophils immediately showed directed chemotaxis and swarming dynamics towards the wound center, while the Cx3cr1-GFP positive macrophages/monocytes migrated at slower speeds and underwent a chemotactic response only after neutrophils had clustered around the tissue lesion (**Video S6**). After 60-75 minutes, macrophages/monocytes had assembled around the neutrophil cluster (**Figure 4A**). To test the instructive role of neutrophils for the secondary recruitment of Cx3cr1-GFP cells to the wound center, we treated neutrophils with neutrophil-depleting antibodies and studied the response of Cx3cr1-GFP cells toward the tissue injury in the absence of neutrophils in the ear skin. In line with our results from the *in vitro* swarming platform, Cx3cr1-GFP cells were still motile and performed random migration in the tissue but lacked directional bias toward the local site of tissue injury (**Figure 4B** and **Video S7**). No reduction of Cx3cr1-GFP cells was observed after neutrophil depletion. Cell migration through the interstitium was tracked in both conditions (**Figures 4C, D**). These observations were confirmed by calculating the swarm size around the site of injury. The area of neutrophils around the injury grew sharply and reached a stable swarm size after 30 min, while monocytes were recruited more gradually over time (**Figure 4E**). In the neutrophil depleted condition, monocytes showed no recruitment toward the site of injury. Additionally, the mean CI of monocytes was significantly higher in the control condition than when neutrophils were depleted (**Figure 4F**).

### Neutrophils and Monocytes Release Monocyte Chemoattractants

**Figure 5:**
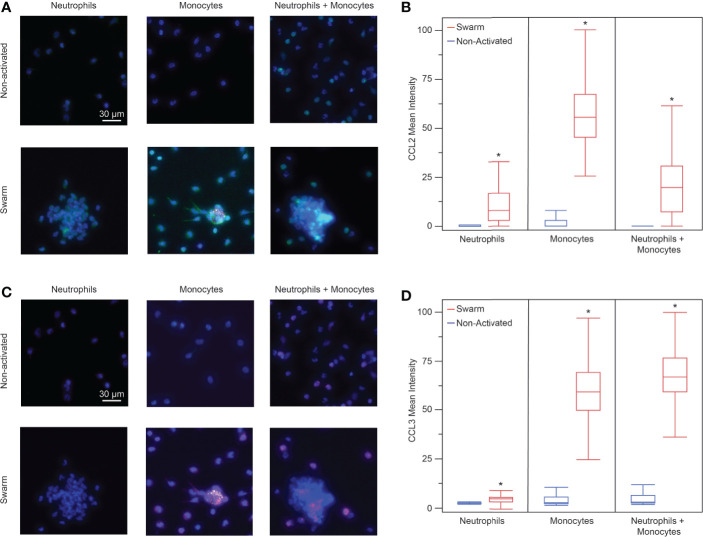
**(A)** Fluorescence images of neutrophils, monocytes, and neutrophils + monocytes (left to right) stained for CCL2. Top: non-activated cells (no bioparticle targets) Bottom: cells on bioparticle targets (blue: nuclei, green: CCL2, yellow: bioparticle target, scale bars: 30 µm) **(B)** Neutrophils, monocytes, and the mixture of neutrophils and monocytes released CCL2 when exposed to bioparticle targets. (mean fluorescence intensity 15.5 ± 38.6, n = 92 cells for neutrophils; 51.4 ± 18.8, n = 970 cells for monocytes; and 18.1 ± 13.8, n = 579 cells for neutrophils + monocytes, Anova, *p < 0.0001 for each condition). There was no significant difference between the non-activated cells and IgG controls (Anova, α = 0.05). The monocyte CCL2 signal was higher than that of the neutrophils or that of the neutrophil + monocyte mixture (Anova, p < 0.001). There was no significant difference between the neutrophil and the neutrophil + monocyte mixture CCL2 signals (Anova, α = 0.05) **(C)** Fluorescence images of neutrophils, monocytes, and neutrophils + monocytes (left to right) stained for CCL3. Top: non-activated cells (no bioparticle targets) Bottom: cells on bioparticle targets (blue: nuclei, magenta: CCL3, yellow: bioparticle target, scale bars: 30 µm) **(D)** Neutrophils, monocytes, and the mixture of neutrophils and monocytes released CCL3 when exposed to bioparticle targets. (mean fluorescence intensity 5.2 ± 2.4, n = 107 cells for neutrophils, and 60.8 ± 13.9, n = 888 cells for monocytes, 66.9 ± 19.8, and n = 456 cells for neutrophils + monocytes, Anova, *p < 0.0004 for each condition). There was no significant difference between the non-activated cells and IgG controls (Anova, α = 0.05). The CCL3 signal for the neutrophil + monocyte mixture was higher than that of the neutrophils or that of the monocytes (Anova, p < 0.001). Additionally, the CCL3 signal from the monocytes was higher than that of the neutrophils (Anova, α = 0.05).

Since monocyte recruitment depends on the presence of neutrophils, we wanted to investigate what chemotactic signals may be involved in this process. We investigated the presence of two proteins known to be involved in monocyte chemotaxis, CCL2 (MCP-1) and CCL3 (MIP-1α), through an *in vitro* immunofluorescence assay at t = 60 min. We found that neutrophils and monocytes both release CCL2 in the presence of bioparticle targets (**Figures 5A, B**). Each condition had significantly higher mean fluorescence than the control condition of non-activated cells incubated on a non-patterned glass slide. The non-activated cells did not have significantly higher fluorescence than the isotype negative controls in any condition. Similarly, each condition in the presence of bioparticle targets had significantly higher CCL3 than the corresponding non-activated control (**Figures 5C, D**). However, the neutrophils had a very low positive CCL3 signal compared to the other two conditions, suggesting that monocytes release more CCL3 than neutrophils. Again, the non-activated controls did not have significant fluorescence when compared to the isotype controls.

### snEVs Cause Monocyte Activation

**Figure 6:**
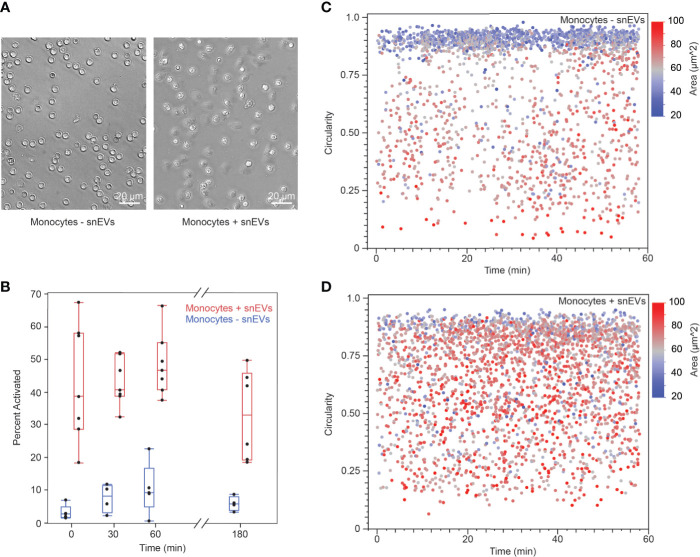
**(A)** Non-activated monocytes (-snEVs, left) had a spherical morphology and did not attach to the substrate. When snEVs were added, the monocytes became activated, developed pseudopods, and adhered to the substrate (scale bars: 20 µm) **(B)** Upon the addition of snEVs, monocytes became activated immediately (42.7% ± 18.0%, N = 7 for t = 0 min). This activation remained approximately constant throughout the first hour after snEV addition but began to decrease by 3 h (54.9% ± 7.9%, 48.3% ± 9.6%, 32.8% ± 13.8%, for t = 30, 60, and 180 min, respectively. p = 0.01 between t = 60 and 180 min). Monocytes without snEVs present exhibited some spontaneous activation in the first 0.5 h of the experiment (p = 0.027 between t = 0 and 30 min), which remained approximately constant throughout the experiment (8.3% ± 4.5%, 10.5% ± 7.8%, 6.0% ± 2.2%, for t = 30, 60, and 180 min, respectively). However, this activation was significantly lower than that of monocytes + snEVs at each time point (p < 0.05). **(C)** Non-activated (- snEV) monocytes were spherical, which could be quantified as high circularity and relatively low 2D area (0.747 ± 0.2318 circularity and 584 ± 137 µm^2^, respectively). **(D)** In the presence of snEVs, monocytes underwent a morphological change, decreasing their circularity and increasing their area (0.655 ± 0.217 circularity and 695 ± 134 µm^2^, p < 0.0001).

To further investigate the mechanisms behind neutrophils’ activation of monocytes, we isolated EVs from swarming neutrophils (snEVs) and added them to monocytes to observe the outcome. We have previously characterized snEVs in detail (7). Characterization of the size distribution and concentration of snEVs can be found in **Figure Supplementary 4**. Monocytes in the presence of snEVs exhibited a clear morphological change (i.e., development of pseudopods and attachment to the substrate) indicative of activation (32, 33). In contrast, monocytes without the addition of snEVs retained a circular (non-activated) morphology (**Figure 6A**). Monocytes were activated within minutes upon the addition of snEVs. This activation persisted throughout the first hour after activation but began to decrease by three hours post-activation (**Figure 6B**). In contrast, the control monocytes demonstrated a slight increase in activation between t=0 and t=30 min and then maintained an approximately constant activation for the remainder of the experiment. The activation of the monocytes both decreased the circularity and increased the 2D area of the monocytes. The control monocytes demonstrated a high circularity and low area throughout the experiment (**Figure 6C**). The monocytes in the presence of snEVs, on the other hand, had a more even distribution of circularity and area throughout the experiment (**Figure 6D**).

## Discussion

Previously, we used our *in vitro* platform to analyze neutrophil swarming (3, 6, 7). Here, we adapted the platform to use PBLs, which enabled simultaneous investigation of the migration of different leukocyte populations. Notably, our system for distinguishing myeloid and lymphoid cells and calculating the myeloid to lymphoid ratio (MLR) provides a simple approximation for the commonly used diagnostic called the neutrophil to lymphocyte ratio (NLR), which is often used as a preliminary predictor of a patient’s response to treatment in various cancers (34–37). In peripheral blood, the neutrophil population has a much wider variation than other myeloid cells (i.e., the normal range of neutrophil counts is 2.09 – 5.97 million cells/mL while the range of monocytes is 0.2 – 0.9 million cells/mL). For this reason, we hypothesize that our myeloid to lymphoid cell ratio (MLR) will correlate to an NLR, though the ratio will be shifted higher to account for the monocyte population. Eosinophils and basophils are present in a low enough concentration to be considered negligible. The mean NLR for a healthy population is around 1.65 ± 1.47 (mean ± SD) (34). The 95% confidence interval (CI) for the lower limit of the NLR in a healthy population is 0.75 – 0.81. The 95% CI for the upper limit is 3.40 – 3.66. The MLR for each of our donors fell within the normal range of the NLR, though one donor neared the upper end of the normal range (3.47) and another neared the lower end (0.86).

Additionally, we were able to analyze the migration patterns of myeloid and lymphoid cells independently. As expected, the myeloid cells, which are innate immune cells, had a much higher response to *S. aureus* bioparticles than the lymphoid cells, which are predominately adaptive immune cells. However, there seemed to be some activation of the adaptive immune cells. Interestingly, the lymphoid cells approached the swarm but rarely entered it. This suggests that there was some activation of the lymphoid cells, even if they did not participate in swarming.

Next, we asked whether we could use our platform to distinguish a healthy, unperturbed immune response from that of a perturbed system. To test this, we added exogenous LTB_4_ to the system. LTB_4_ is known as one of the primary chemoattractants that neutrophils release during swarming (3). Interestingly, exogenous LTB_4_ impacted myeloid cell migration but had no significant effect on lymphoid cell migration. This suggests not only that LTB_4_ did not directly affect lymphoid cells, but also that the altered myeloid cell response did not impact the subsequent lymphoid cell response. Our data suggest that we can use our platform to identify immune cells that act abnormally. Since the LTB_4_ only impacted myeloid cell migration, we also hypothesize that we could distinguish an altered myeloid cell response from an altered lymphoid cell state to investigate the nature of a perturbation (i.e., whether it impacts a certain cell type).

Since our platform elicited a more pronounced response among the myeloid cell population, we wanted to further investigate the myeloid cell population. The main myeloid cells in the peripheral blood are neutrophils and monocytes. In the peripheral blood, neutrophils are on average 89% of myeloid cells (31). However, we increased the monocyte concentration to a 1:1 ratio to make our results statistically viable. Unsurprisingly, neutrophils swarmed regardless of the presence of the monocytes. The monocytes did not significantly impact neutrophil migration. However, monocytes only exhibited swarming-like behavior in the presence of neutrophils. This suggests that neutrophil signals are essential for the activation of monocytes. We confirmed these results using *in vivo* experiments, which demonstrated that monocyte recruitment succeeded neutrophil recruitment in a control condition, but no monocyte recruitment occurred when neutrophils were depleted. These results are supported by a previous *in vivo* study that also observed that monocytes were only recruited to an inflammation site when neutrophil swarms were present, and went on to suggest that monocytes may participate in controlling the growth of a neutrophil swarm (38). Additionally, both neutrophils and monocytes released the monocyte chemoattractants CCL2 and CCL3 during swarming. It is interesting to note the presence of these chemoattractant markers in the monocyte-only conditions where swarming does not occur. This suggests either that the gradient created by these chemoattractants was not strong enough to induce directed monocyte migration or that the gradient was not formed quickly enough for us to capture it in our experiments.

Since CCL2 and CCL3 were released by monocytes, this suggests that the difference in monocyte activation and migration did not occur *via* these free mediators. We cultured and purified snEVs as we did in a previous study (7). We added snEVs to non-activated monocytes to measure monocyte activation through alteration of cell morphology. Our data overwhelmingly demonstrated that snEVs caused monocyte activation, while non-activated monocyte controls remained non-activated. Activated monocytes exhibited an increase in 2D surface area and a decrease in circularity. This demonstrates that snEVs play a role in monocyte activation, which is corroborated by a recent research study (39).

In conclusion, we established that our platform can be used to distinguish the migration profiles of various cell types. Our platform was able to detect differences between the migratory behaviors of myeloid cells when perturbed with exogenous LTB_4_. We have found that neutrophils are essential for monocyte recruitment, and one way this activation can be achieved is through snEVs. Our data suggest that neutrophils, which are the first responders of the immune system, are also essential in starting the activation of other immune cells that follow later in the immune cascade.

## Data Availability Statement

The original contributions presented in the study are included in the article/**Supplementary Material**. Further inquiries can be directed to the corresponding author.

## Ethics Statement

The studies involving human participants were reviewed and approved by Biomedical Sciences Committee institutional review board (IRB #2018H0268) at The Ohio State University. The patients/participants provided their written informed consent to participate in this study. The animal study was reviewed and approved by NIAID Animal Care and Use Committee (National Institutes of Health).

## Author Contributions

NW and ER contributed to the study design, interpretation of results, and manuscript drafting. NW designed, performed, and analyzed *in vitro* experiments. JZ designed and performed the EV purification and concentration. XR and LN assisted with the design of laboratory methods and experiments. RG and TL designed and performed the *in vivo* experiments. ER oversaw the project. All authors contributed to the article and approved the submitted version.

## Funding

The authors were supported by funding from the Chan Zuckerberg Initiative (CZI) grant # 2020-217723 (ER and TL). The funder was not involved in the study design, collection, analysis, interpretation of data, the writing of this article or the decision to submit it for publication. ER was supported by William G. Lowrie Department of Chemical and Biomolecular Engineering and the Comprehensive Cancer Center at The Ohio State University (ER). Data presented in this report came from images processed using Imaris x64 (ver. 9.3.0 Bitplane) available at the Campus Microscopy and Imaging Facility, The Ohio State University. This facility is supported in part by grant P30 CA016058, National Cancer Institute, Bethesda, MD.

## Conflict of Interest

The authors declare that the research was conducted in the absence of any commercial or financial relationships that could be construed as a potential conflict of interest.
