# Supplementary material for: Analyzing Inter-Leukocyte Communication and Migration In Vitro: Neutrophils Play an Essential Role in Monocyte Activation During Swarming
Source: Front Immunol. 2021 May 12;12:671546. doi: 10.3389/fimmu.2021.671546 (PMC8152805; doi:10.3389/fimmu.2021.671546)
Supplement: Supplementary file 1 [file DataSheet_1.docx]

**Analyzing Inter-Leukocyte Communication and Migration *in vitro*: Neutrophils Play an Essential Role in Monocyte Activation during Swarming**

**Nicole Walters,^1^ Jingjing Zhang,^1^ Xilal Rima,^1^ Luong T. H. Nguyen,^1^ Ronald N. Germain,^2^ Tim Lӓmmermann,^2,3^ Eduardo Reátegui^1,4*^**

^1^ William G. Lowrie Department of Chemical and Biomolecular Engineering, The Ohio State University, Columbus OH 43210

^2^ Laboratory of Immune System Biology, National Institute of Allergy and Infectious Diseases, National Institutes of Health, Bethesda, MD, USA.

^3^ Max Planck Institute of Immunobiology and Epigenetics, Freiburg, Germany.

^4^ Comprehensive Cancer Center, The Ohio State University, Columbus, OH 43210

^*^Corresponding author: reategui.8@osu.edu

**Supplementary Information**

**Video S1.** PBLs migration toward *S. aureus* bioparticles. Cell tracks are shown according to myeloid / lymphoid classification. (**Fluorescence:** yellow: *S. aureus* bioparticles, cyan: PBL nuclei, red: PBL cytoplasm. **Cell tracks:** green: myeloid cells, purple: lymphoid cells.)

**Video S2.** PBL migration toward S. aureus bioparticles in the presence of 100 nM LTB_4_, Cell tracks are shown according to myeloid / lymphoid classification. (**Fluorescence:** yellow: *S. aureus* bioparticles, cyan: PBL nuclei, red: PBL cytoplasm. **Cell tracks:** green: myeloid cells, purple: lymphoid cells.)

**Video S3.** Neutrophil migration toward *S. aureus* bioparticles. (**Fluorescence:** yellow: *S. aureus* bioparticles, green: neutrophils. **Acquisition time:** 0 – 3 h.)

**Video S4.** Monocytes do not migrate toward *S. aureus* bioparticles. Some monocytes reach the bioparticle target through random migration. Upon reaching the target, the cells become activated and adhere to the target. No directed monocyte migration is observed. (**Fluorescence:** yellow: *S. aureus* bioparticles, green: neutrophils, magenta: monocytes. **Acquisition time:** 0 – 3 h.)

**Video S5.** Neutrophils and monocytes migrate toward *S. aureus* bioparticles when added together. (**Fluorescence:** yellow: *S. aureus* bioparticles, green: neutrophils, magenta: monocytes. **Acquisition time:** 0 – 3 h)

**Video S6.** Endogenous neutrophil and macrophage/monocyte behavior around a small focal, laser-induced tissue injury in *DsRed^+/-^ Cx3cr1^gfp/gfp^ Tyr^c-2J/c-2J^* mice (macrophages/monocytes in green, stroma and neutrophils in red).

**Video S7.** Macrophage/monocyte behavior in neutrophil-depleted (anti-Gr1) mice around a small focal, laser-induced tissue injury in *DsRed^+/-^ Cx3cr1^gfp/gfp^ Tyr^c-2J/c-2J^* mice (macrophages/monocytes in green, stroma in red).


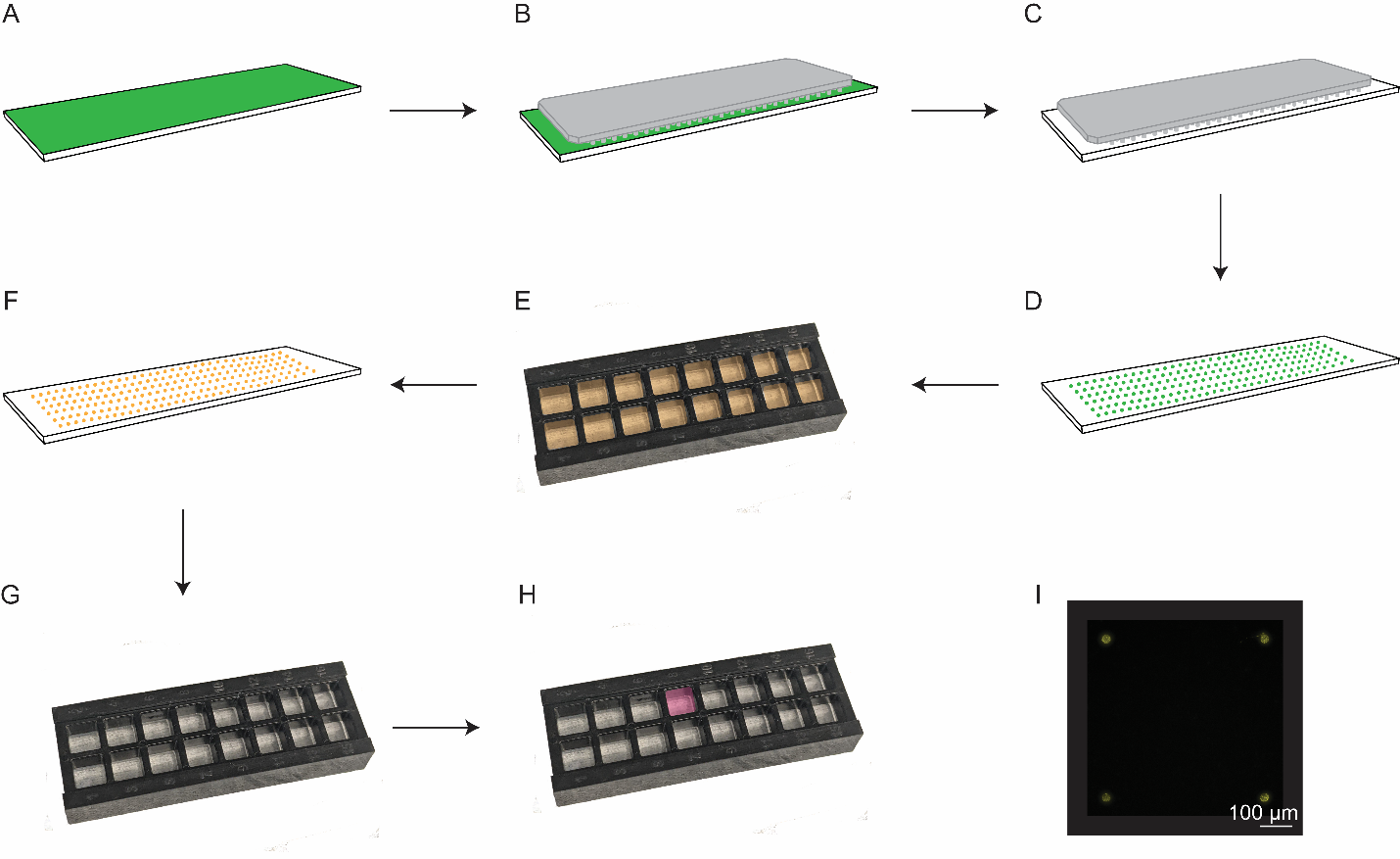


**Figure S1.** Diagram of device fabrication. **A.** A layer of a cationic polymer, Zetag, was spun onto a clean glass slide. **B.** A PDMS stamp with an array of circular posts (30-µm diameter, 500-µm center-to-center spacing, and 40-µm tall) was incubated on the Zetag layer for 20 min. **C.** The coated stamp was then placed onto a clean glass slide for 10 min to allow the Zetag layer to transfer. **D.** The stamp was removed, leaving an array of 30-µm diameter Zetag spots. **E.** A 16-well chamber was secured to the slide, and a solution of *Staphylococcus aureus* (Wood strain without protein A) bioparticles conjugated to Alexa Fluor 594 was incubated in the wells for 20 min. Since Zetag is positively charged, the negatively charged bioparticles adhere to the Zetag spots via electrostatic interaction. **F.** The 16-well chamber was removed, and a strong stream of DI water was used to remove excess bioparticles. The prepared device can be stored for up to 3 months at 4 °C in a dust-free environment. **G.** On the day of the experiment, the 16-well chamber was built on the device and the surface of selected wells was coated with fibronectin in FBS. **H.** The prepared cell solution is added to the selected wells to start the experiment. Up to 16 experimental conditions can be run in parallel. **I.** A fluorescent image of a prepared bioparticle array. The bioparticle clusters are 30 µm in diameter and spaced 500 µm apart. Initially, no chemoattractant gradient is present. Upon reaching the clusters, neutrophils become activated by the pathogen-associated molecular patterns (PAMPs) on the bioparticles and generate a chemoattractant gradient that directs cell migration.

**
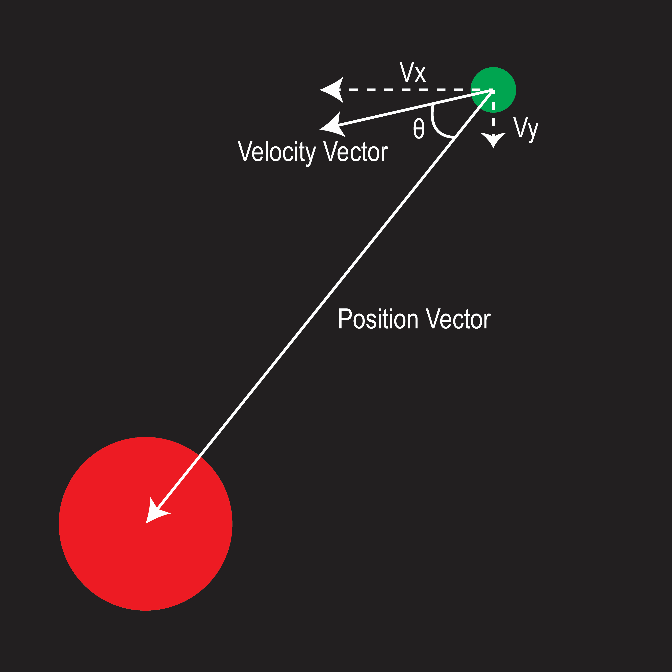
**

**Figure S2.** Diagram of chemotactic index (CI) calculation. The velocity vector was determined as the hypotenuse of the velocity in the X direction (Vx) and the velocity in the Y direction (Vy) exported from Imaris. The position vector was calculated using the X, Y positions of the cell (green) and bioparticle target (red) exported from Imaris. The angle θ was measured as the angle between the vectors. Then, CI = cos(θ).


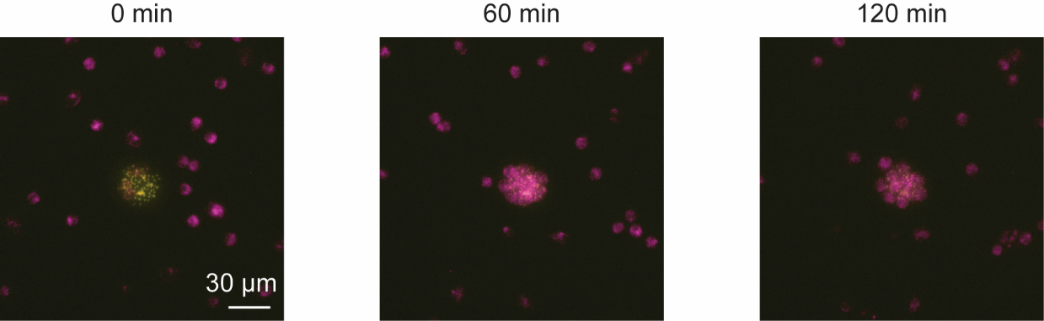


**Figure S3.** Monocyte behavior varied from donor to donor. One donor experienced monocyte accumulation around the bioparticle targets without the presence of neutrophils.


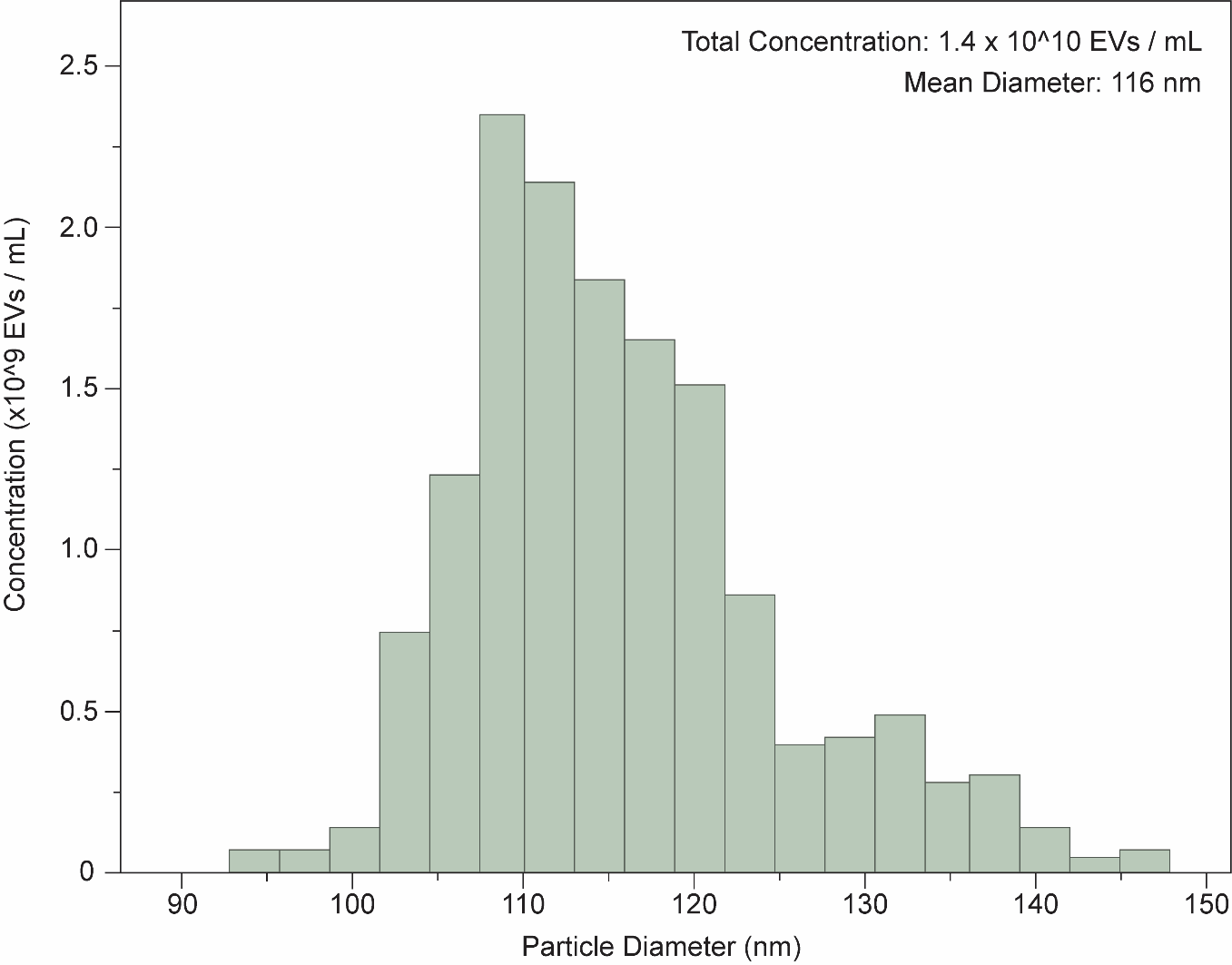


**Figure S4.** snEV Characterization. TRPS was used to measure the concentration and diameter of EVs released by swarming neutrophils after purification and concentration. (Concentration: 1.4x10^10^ snEVs / mL, mean diameter: 116 ± 45 nm).

**Supplementary Table 1.** Myeloid and lymphoid cell counts used to calculate the MLR.

|  | Myeloid Cell Count | Lymphoid Cell Count | Myeloid to Lymphoid Ratio (MLR) |
| --- | --- | --- | --- |
| Donor 1 | 1706 | 492 | 3.46748 |
| Donor 2 | 359 | 233 | 1.540773 |
| Donor 3 | 976 | 1131 | 0.862953 |
| Donor 4 | 981 | 923 | 1.062839 |
| Donor 5 | 1273 | 1139 | 1.117647 |
